# Growth of ‘W’ doped molybdenum disulfide on graphene transferred molybdenum substrate

**DOI:** 10.1038/s41598-018-25796-9

**Published:** 2018-05-09

**Authors:** Vijayshankar Asokan, Dancheng Zhu, Wei Huang, Hulian Wang, Wandong Gao, Ze Zhang, Chuanhong Jin

**Affiliations:** 1 State Key Laboratory of Silicon Materials, School of Materials Science and Engineering, Zhejiang University, Hangzhou, Zhejiang, 310027 China; 2 Present Address: Environmental Inorganic Chemistry, Department of Chemistry and Chemical Engineering, Chalmers University of Technology, Gothenburg, 41296 Sweden

## Abstract

In the present study, a novel method has been carried out to grow tungsten (W) doped molybdenum disulfide (MoS_2_) on the graphene transferred TEM grid in a chemical vapor deposition (CVD) setup. Tungsten trioxide (WO_3_) has been used as a source for ‘W’ while ‘Mo’ has been derived from Mo based substrate. Different experimental parameters were used in this experiment. Higher gas flow rate decreases the size of the sample flake and on other side increases the dopant concentrations. The interaction mechanism between Mo, S, W and oxygen (O) have been explored. The influence of oxygen seems to be not avoidable completely which also imposes effective growth condition for the reaction of Mo with incoming sulfur atoms. The difference in the migration energies of Mo, WO_3_, S clusters on the graphene and the higher reactivity of Mo clusters over other possibly formed atomic clusters on the graphene leads to the growth of W doped MoS_2_ monolayers. Formation of MoS_2_ monolayer and the nature of edge doping of ‘W’ is explained well with the crystal model using underlying nucleation principles. We believe our result provide a special route to prepare W doped MoS_2_ on graphene substrate in the future.

## Introduction

Recent research of two-dimensional (2D) transition metal dichalcogenides (TMDs) materials, and its successful integration into the devices opens up a new direction in applications^1–20^. Molybdenum (Mo) and tungsten (W) based dichalcogenides are two such representatives of well-defined family of structurally and chemically ordered 2D compounds. The basic crystal structure of 2D TMDs materials consists of hexagonally packed atomic layers, with chalcogenide–metal–chalcogenide arrangements in the trigonal prismatic or octahedral coordination^21^.

Alloying is widely used due to its ability to accurately controlling doping concentrations which determines the physical and chemical properties of materials^19,22^ and hence comparatively better than other band gap engineering processes (such as strain, chemical functionalization, etc.). Isoelectronic doping can be also useful in suppressing the detrimental effect of defects created during the growth of TMDs monolayers and hence increases photoluminescence efficiency which paves the way for the TMDs materials to find its applications in opto-electronic devices^22,23^.

In present research, we have considered W-doped molybdenum disulfide (MoS_2_). Tungsten doping into the MoS_2_ in a continuum compositional range Mo_x_W_x−1_S_2_ for 0 ≤ x ≤ 1 is energetically favorable mainly due to two reasons: 1) both MoS_2_ and WS_2_ possess the same hexagonal parent structure and 2) a comparable Shannon Prewitt crystal radius, 0.790 Å (Mo^4+^) vs 0.800 Å (W^4+^) and a well-matched lattice constant^19,24^. The monolayers of MoS_2_ are intrinsically n-type, while WS_2_ and WSe_2_ are intrinsically p-type, and their energies are mainly contributed by their d-orbitals of tungsten. Since both Mo and W possess different d-orbitals, the band gap engineering can be done by the doping of W atoms into MoS_2_^22,23^.

Even though, W-doped MoS_2_ have been successfully obtained by mechanical exfoliation, it is found to be inappropriate in the practical implementation into devices. On the other side, chemical vapor deposition (CVD) found to be successful in the growth of large area MoS_2_ monolayers. In a traditional CVD setup, the sulfur (S) powder is mostly used as a reductant source. The ‘S’ vapor partially reduce volatile MoO_3−x_/MoO_2−x_/WO_3−x_, and increases the probability of MoS_2_ or WS_2_ monolayer formation depends on the metal precursors used^25–28^. The difference in their respective vapor pressures of Mo and W precursors establish the difficulties in achieving the controlled supply of ‘W’ atoms for the synthesis of W doped MoS_2_ alloy and hence extra care must be taken into an account^22^.

**Figure 1:**
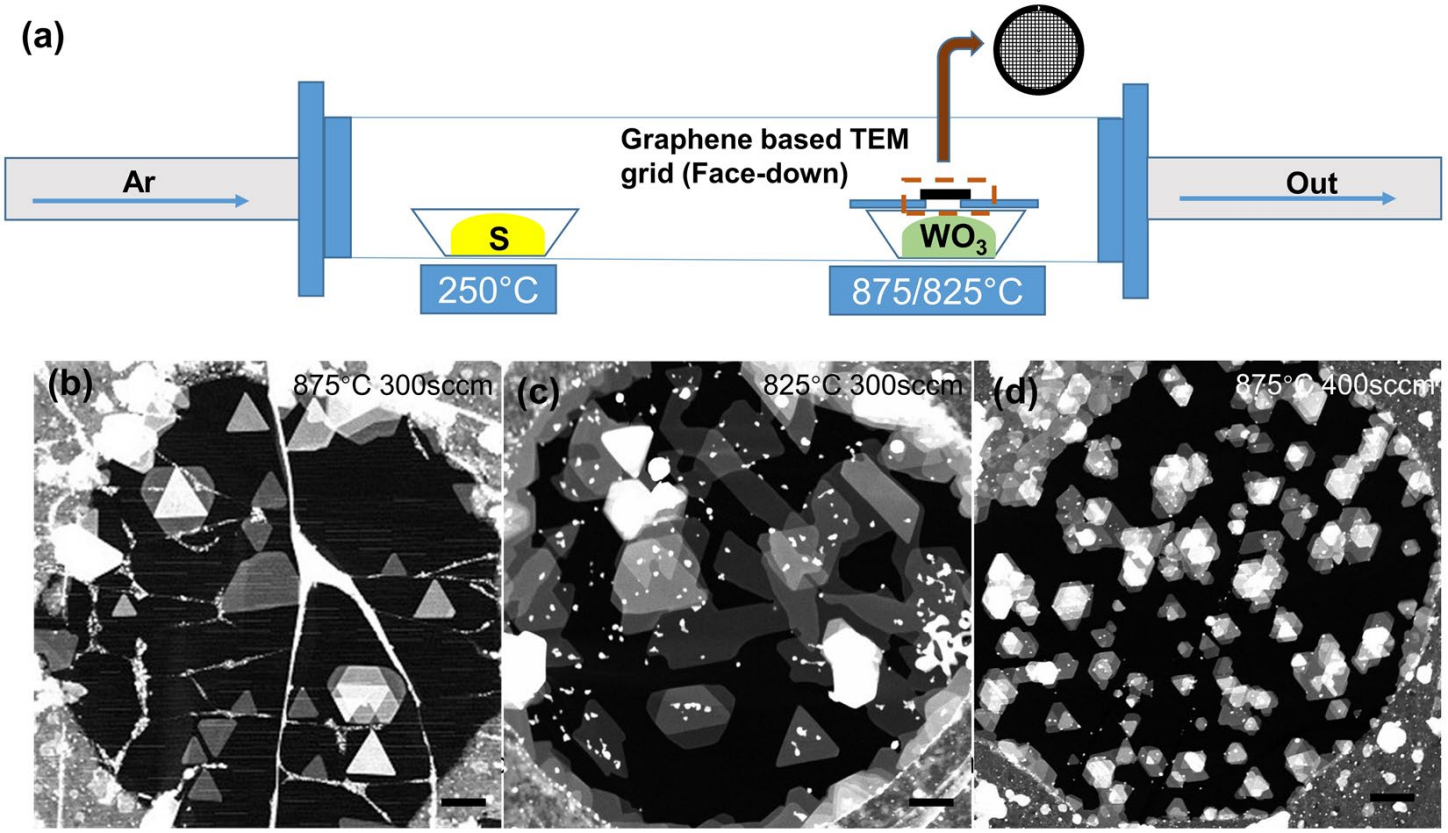
(**a**) Schematic of chemical vapor deposition experimental set-up. Typical low magnification ADF-STEM images of (**b**) MWS-1a, (**c**) MWS-1b and (**d**) MWS-1c. Scale bar: 200 nm.

Previous researches have been conducted using Mo solid thin films prepared by thermal evaporation^29,30^, electron beam evaporation^31,32^, sputtering^33^ and then sulfurization continued either in the second step of experiment or in a specially designed system^33^, in all the cases the resulting MoS_2_ layers were dependent on the thickness of deposited thin films and so far, produced only MoS_2_ monolayers without any possible doping. In this work, the sulfurization of Mo and then doping of W into MoS_2_ on the graphene have been carried out in a single and simple CVD set-up (refer Fig. 1a). We have used Mo based substrate as a source for ‘Mo’ and WO_3_ powder as a source for ‘W’. MoS_2_ monolayers are expected to grow first in this process, due to the higher chance for Mo to react with incoming ‘S’ atoms compared to ‘W’ atoms, and hence, ‘W’ atoms reach after the formation of MoS_2_ monolayer will find its place as dopant atoms at Mo lattice site. Direct observation of such alloy structure at the atomic scale is essential to understand and to further control the wide range of its behavior, and hence, graphene has been chosen as a substrate and placed on TEM grid to prevent the loss of small atomic clusters during the traditional transfer process when it grows on SiO_2_, or sapphire or mica^34^. In addition, the formation of graphene and transition metal doped heterostructures is very interesting, owing to the combination of high mobility and transparency of graphene and the exceptional optical properties of MoS_2_^18,35^.

## Results and Discussions

To verify the possibilities of ‘Mo’ to be derived from Mo based substrate and to evaluate the interaction mechanism of Mo with S and Mo with W, the experiments with different parameters (refer Table 1) were conducted using typical CVD set-up shown in Fig. 1a. A typical low magnification annular dark-field (ADF) scanning transmission electron microscope (STEM) images of MWS-1a, MWS-1b and MWS-1c are displayed in Fig. 1(b–d), respectively. The flakes in MWS-1a (Fig. 1b) are identified to grown at a scale of 70–200 nm, and most of them are in uniform orientation.

MWS1-b (Fig. 1c) contains the flakes around 75–250 nm. MWS-1b contains nanostrips and other polygons, while MWS-1a contains mostly with triangles while other polygons are rarely found. Transformation of triangular, hexagonal flakes to a layer with different morphologies through the collision of neighboring structures usually observed in 2D materials growth^25,36^.

MWS-1c (Fig. 1d) shows it contains triangles in a large distribution, while almost formed in multi-layer or layer over layer growth. The flakes are grown at a scale of 50–80 nm, much smaller compared to MWS1-a. Flow rate of carrier gas, Ar 400 sccm, reduces the growth propagation while increases the nucleation density (Fig. 1d), compared to the flow rate, Ar 300 sccm (Fig. 1b). The growth temperature 875 °C seems to impose the best condition for the evolution of triangles, however, the argon flow rate decides the high-quality growth.

Growth of monolayers on graphene also seems to be influenced by the underlying substrate and on the graphene synthesis techniques^37^. In all our present experiments, the underlying substrate is TEM grids and the graphene for all these experiments were transferred to TEM grids grown on the same copper foil, and hence there were no variation of influence based on the substrate principles.

At relatively lower temperature, that is at 825 °C, for the sample MWS-1b (Fig. 1c), the sulfurization in the initial period is difficult, leading to the variation of Mo:S ratio and the MoS_2_ becomes unsaturated^38^. Mo-terminated zig-zag (Mo-zz) will always grow faster than S-terminated zig-zag (S-zz)^39–41^, and the relatively lower temperature would increases the differences in the rate of edge growth, and leads to the other morphologies^38^ than the triangles and hexagons, in this case its nanostrips (Fig. 1c). At the same time, with the increase in flow rate of carrier gas, S-concentration is relatively higher^40^, and with the variation of Mo:S ratio, with S-rich environment, the rate of growth between two edges, Mo-zz and S-zz would be small, results in a small triangle with truncated shape^39,41^.

On an average, 20–25 flakes have been counted to every 5 μm circumference region of TEM grid in MWS-1a samples, while MWS-1b sample contains less than 10 flakes, rest is filled with polygons and MWS1-c sample contains more than 75–100 flakes, however identifying individual flakes are hard as it contains mostly a layer over layer growth.

**Figure 2:**
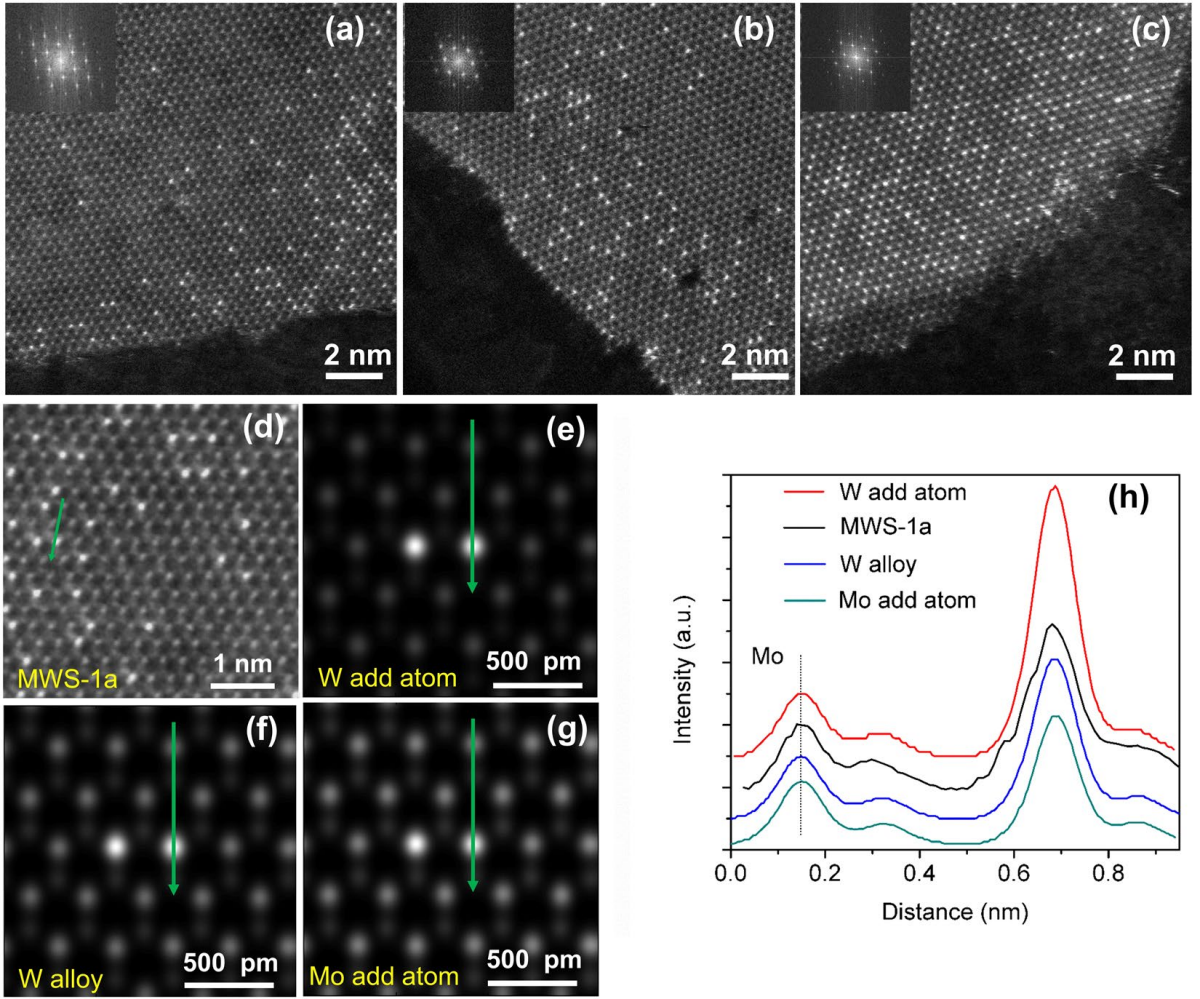
A typical atomic-scale ADF-STEM images of MWS-1a, MWS-1b and MWS-1c are displayed in (**a**), (**b**) and (**c**), respectively. MWS-1c contains highly concentrated W doping at the edges of MoS_2_ layers compared to MWS-1a and MWS1-b. (**d**) The experimental image of a selected region of W doped MoS_2_ structure within MWS-1a and the corresponding line profile. (**e**–**h**) The simulation images and the corresponding line profiles. The intensity of line profile in (**d**) matches with the line profile in (**f**) confirms that the W atoms are doped into the MoS_2_ monolayer.

An atomic-scale resolution ADF-STEM images of MWS-1a, MWS-1b and MWS-1c are displayed in Fig. 2(a–c). All three images show the distribution of W doping within a monolayer, effective doping around the corners of a flake, while the interior part without any effective doping. The experimental image (Fig. 2d) of a selected region of W doped MoS_2_ structure within MWS-1a (Fig. 2a). And the corresponding line profile together with simulation data (Fig. 2(e–g)). The intensity of line profile in Fig. 2d matches with the line profile in Fig. 2f confirms that the W atoms are doped into the MoS_2_ monolayer. During the STEM characterization, it is already evaluated that there is no displacement of atoms of higher intensity confirms those atoms are alloy atoms and not probably ‘Mo’ or ‘W’ add atoms. QSTEM simulation^42^ have also been employed to validate it. Intensity profile of marked region (green arrow) in Fig. 2d illustrating MWS1-a sample matches well with Fig. 2g intensity profile, confirms that those bright atoms are W alloy into MoS_2_ monolayers.

**Figure 3:**
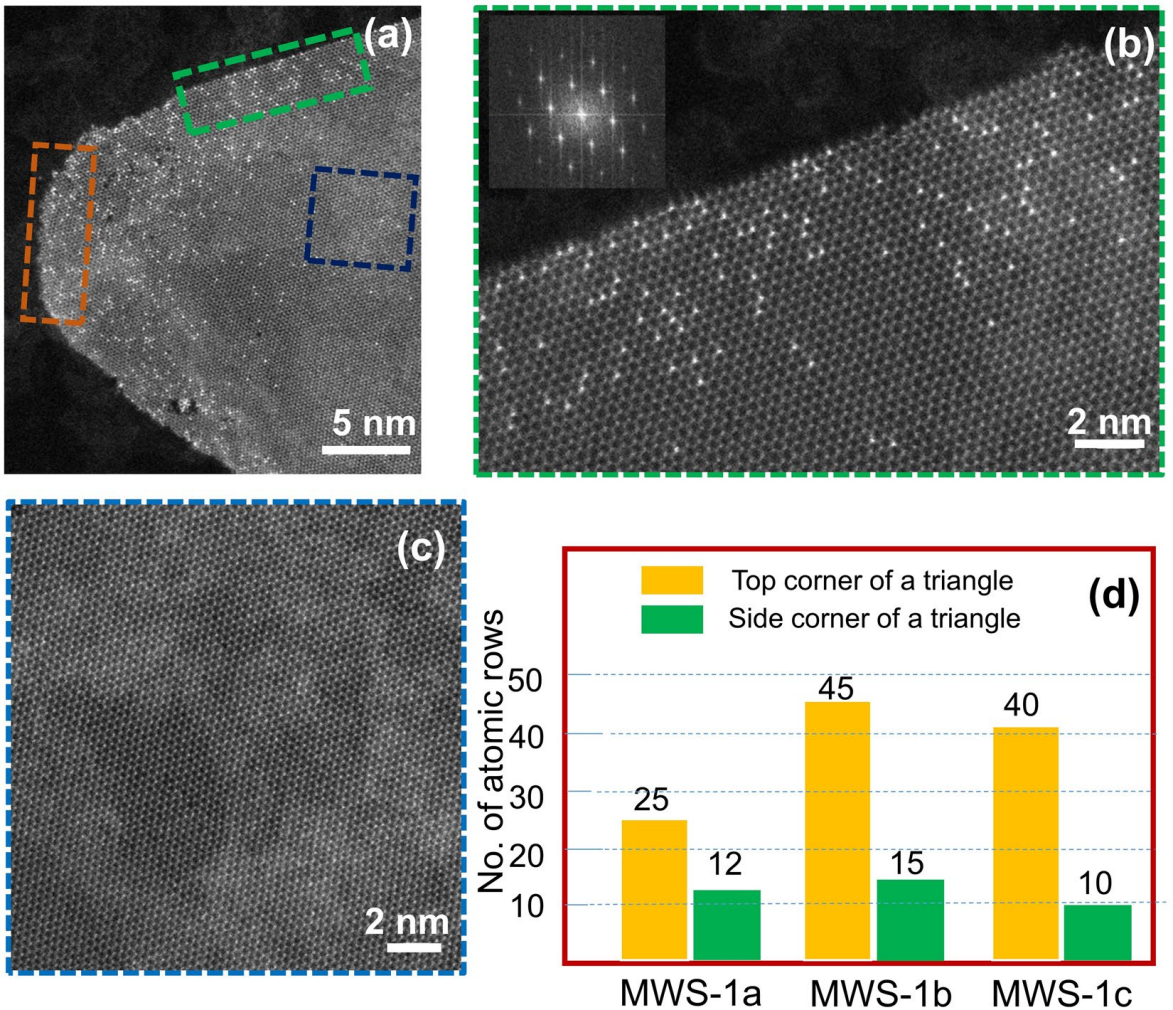
(**a**) An atomic-scale resolution ADF-STEM images of MWS-1b is displayed and an approximate area within green rectangular and blue square regions marked are illustrated in (**b**) and (**c**), respectively. Orange rectangle also depicts the region of top-edge doping, and the green rectangle depicts the region of side-edge doping. Inset in (**b**) display a FFT of the shown region which portrays a single crystalline nature of a monolayer, all three shows the distribution of W doping within a monolayer, effective doping around the corners of a flake, while the interior part (**c**) portrays with no dopants. (**d**) Histogram of the amount of the top and side corner atoms.

An atomic-scale resolution ADF-STEM images of MWS-1b is displayed in Fig. 3a and an approximate area within green rectangular and blue square regions marked in Fig. 3a are illustrated in Fig. 3b and c respectively. Inset in Fig. 3b display a FFT of the shown region which portrays a single crystalline nature of a monolayer, all three shows the distribution of W doping within a monolayer, effective doping around the corners of a flake, while the interior part without any effective doping. From the analysis of 25 atomic-scale images of all the three samples (MWS-1a, MWS-1b and MWS-1c), it is calculated that at an average (Fig. 3d). W dopant atoms are distributed to 25 atomic rows on the top edge of flakes in MWS-1a samples (marked in orange rectangle) while the dopant atom distributions are up to 12 atomic rows on the side edges of flakes (marked in green rectangle). In samples MWS-1b and MWS-1c, dopant atoms are distributed to 45 and 40 atomic rows, respectively, on the top edges of flakes, while on side edges of flakes, the distribution is up to 15 and 10, respectively. Hence, higher carrier gas flow rate not only increased the dopant concentration, also increased number of layers, while mostly of layer-over layer formation.

**Figure 4:**
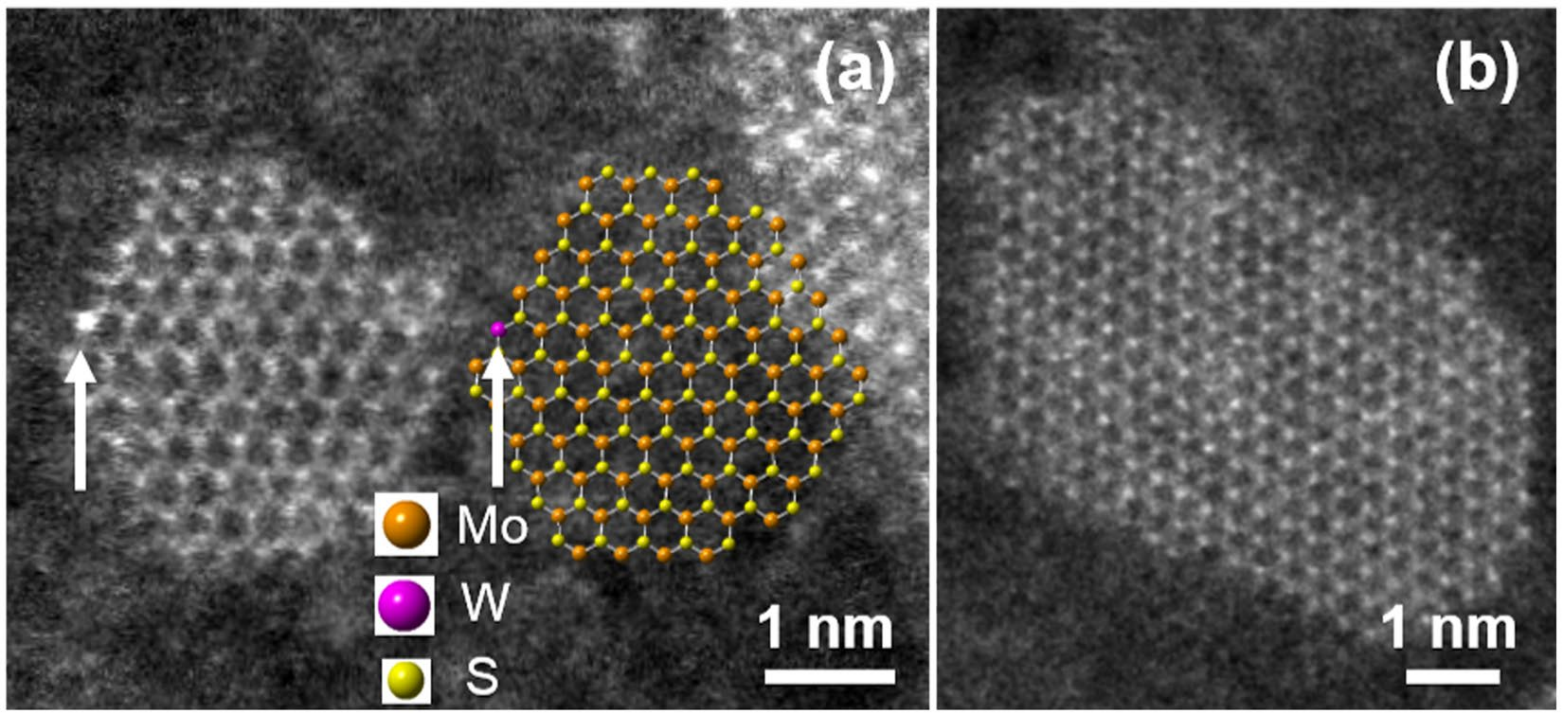
(**a**,**b**) An atomic-scale ADF-STEM image and crystal model (right side) of two small atomic clusters of MoS_2_ in MWS-1c. Arrow pointing the region in first cluster (**a**) depicts a single W atom doping.

Atomic-scale ADF-STEM images and crystal model of some small atomic clusters of MoS_2_ (MWS-1c) are displayed in Fig. 4(a,b). Of more than 30 samples we checked, most samples in small size are MoS_2_ monolayer clusters without W atom doping. Only one W atom was found which was marked in Fig. 4a (Arrowed). This result confirms that in this growth process, MoS_2_ was prepared first. The formation of MoS_2_ nanoflakes and monolayers are also possible in the growth temperature range of 700–750 °C, as evident from the reported elsewhere^13,41^, we suspect these small clusters might be formed during the cooling stage, at which point, Mo and S are still energetically active species to react and grow further, while W atoms does not have a chance to find its energetically active position in the already formed MoS_2_ structures.

To further elucidate the contribution of Mo atoms (clusters) derived from Mo-based substrate into the reaction and to discuss the role of oxygen in the monolayer formation mechanism, low magnification ADF-STEM images and Energy dispersive X-ray spectroscopy (EDS) mapping of as-synthesized MoS (Fig. S1) and MoWO (Fig. S2). MoS sample possess irregular and small sized particles (Fig. S1a), while MoWO sample consists mostly of regular shaped bright particles (Fig. S2a). Sulfurization of Mo/TEM grid without WO_3_ precursor does not produce any MoS_2_ monolayer flakes, suggests partial pressure environment formed by the addition of W-O_(x)_/WS_x_O_(3−x)_ is necessary for optimum experimental condition to grow MoS_2_. EDS mappings (refer Fig. S2(b–e) for MoS and Fig. S2(b–e) for MoWO) shows the common trend in both the samples, Mo signals are stronger in the core. S and W signals for respective MoS and MoWO samples are detected in the larger area, extended to outer shell.

**Figure 5:**
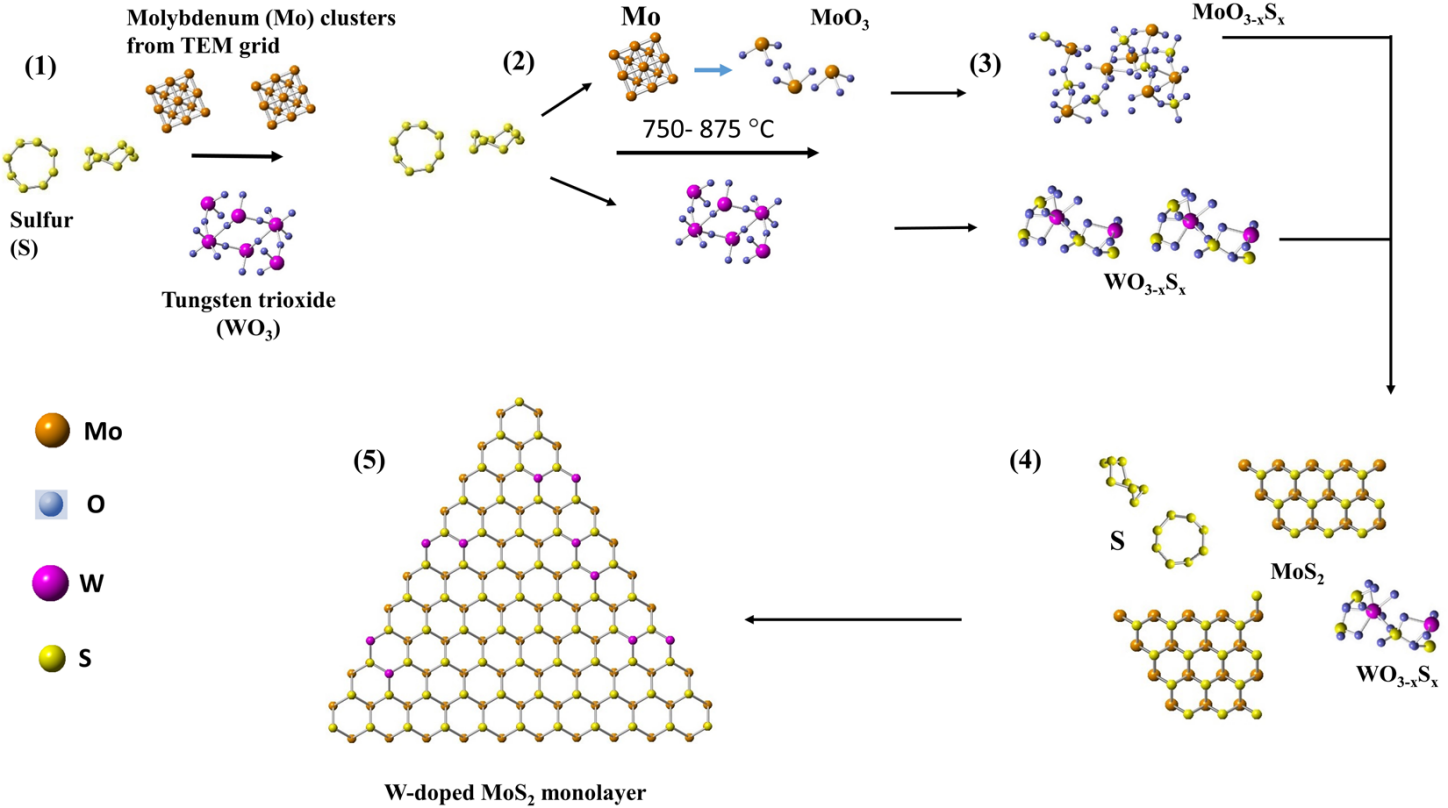
The general growth mechanism of W- doped MoS_2_.

According to these results, we could discuss the microscopic growth process of W doped MoS_2_ (Fig. 5). The availability of ‘O’ may play an important role during this special CVD growth process. Oxygen (O) in these reactions are probably derived from the residue happened to form during chemical-wet transfer of graphene into TEM grid from copper substrate, residual atmosphere and the oxidation of molybdenum. In the traditional CVD process, the excess oxygen may cause the etching of MoS_2_^43^ and play a major role in the particles or fullerene structure formation as reported earlier in few works^41,44^. In this process, more oxygen is required to form MoO_x_ clusters first.

After sulfurization (in the case of MoS sample), Mo clusters possibly forms an amorphous alloy which possess octahedral or tetrahedral co-ordination^45,46^. Due to this complexity in its structure, it requires large energy to change its geometry to evolve as two-dimensional hexagonal network^45,47^. Speaking in terms of partial pressure environments, higher S partial pressure environment would lead to the fullerene structure, lower S partial pressure leads to oxy-sulfide particles^48^. In this case, promoter such as WO_3_ in its reduced form W-O_(x)_/WS_x_O_(3−x)_ probably needed to reduce the complex structure as in the case of MoS and provides suitable S partial pressure environment to form two-dimensional hexagonal network of MoS_2_.

In step 2, at the high temperatures between 825–875 °C, Mo get oxidized and forms thermally active MoO_3_ structures together with Mo clusters which is not oxidized. Due to the differences in the surface free energy of graphene (around 46.7 mJ/m^2^)^49^ and Mo (around 2.92–3.34 J/m^2^)^50^, metal atoms migrating on graphene is expected to form molecular clusters on graphene^51,52^, which is more reactive with sulfur and oxygen than the Mo atoms and W-O_(x)_ clusters, and hence readily reacts with the incoming sulfur atoms (Step 3)^31,33^. The reductant source ‘S’ vapor involves simultaneously in reducing WO_3_. Owing to its lower vapor pressure, an intermediate compound WS_x_O_(3−x)_ is expected to form (Step 3)^53^.

In Fig. 5, WS_x_O_(3−x)_ is depicted in its simple cluster form WSO_2_ for an easy understanding. MoS_2_ monolayer in its triangular form starts to grow first and the intermediate product WS_x_O_(3−x)_, together with few individual Mo and S clusters are at the near-edge site of MoS_2_ (Step 4). In the following reaction step, WS_x_O_(3−x)_ reduces and W atoms replace Mo at its lattice sites because of the shortage of Mo due to the reduced diffusion of Mo atoms (Step 5)^54^.

In summary, W-doped MoS_2_ monolayer structures on graphene have been synthesized using Mo atoms (clusters) derived from Mo based substrate. Comparing to the growth temperatures, 875 °C and 825 °C, the former case provides high-quality and uniform triangular MoS_2_ monolayers with less concentrated W doping, while in the latter case, even though the concentration of W doping among the individual triangular monolayer structures is similar as in the case of former one, merged monolayer structures with irregular shape have been found in majority. Nucleation density is large at lower temperature, however, the probability of finding individual triangles is lesser. The size of flake grown in lower temperature experiment is also larger. Variation in carrier gas flow conditions (400 sccm) have been done to verify the tuning possibilities of dopant metal atoms into the MoS_2_ monolayers and found that the dopant atoms are distributed up to 60 atomic rows, while in other experiments with carrier gas flow condition of 300 sccm, the dopant atoms distribution is up to maximum of 40 atomic rows (in both temperatures, 875 °C and 825 °C). Higher gas flow rate also decreases the size of the flakes and on other side increased the dopant concentrations. The interaction mechanism between Mo, S, W and O on the graphene and the formation of MoS_2_ monolayer and the nature of edge doping of ‘W’ have been briefly discussed. The influence of oxygen seems to be not avoidable completely and also impose effective growth condition for the reaction of Mo with incoming sulfur atoms. Doping is effective in all the growth conditions and in all structures irrespective of the morphologies, from small clusters to nanostrips indicate the successful nature of doping mechanism. This method of using graphene transferred TEM grid substrate could give us a new idea to prepare TMDs alloys (Re, Ta) in the future.

## Methods

### Sample preparation

Graphene films grown on polycrystalline copper foils were transferred onto molybdenum (**Mo**) based TEM grids using a standard PMMA-assisted wet-chemistry transfer process. The TEM grids with graphene facing downwards were mounted onto a home-built ceramic carrier and 1 mg of Tungsten trioxide (**WO**_**3**_) precursors (Sigma-Aldrich, 99%) placed on the silicon wafer. Quartz boat carrying silicon wafer and TEM grid at its center were loaded into the CVD chamber.

Within a typical CVD process, the furnace was firstly heated to 300 °C in 10 mins and hold for additional 10 mins, and then increased to its growth temperature, 825 °C/875 °C in 45 mins. Just 2 mins before the main CVD furnace reaches its growth temperature, 300 mg of sulfur (**S**) source (Aladdin, 99.999%) were heated (reaches 250 °C in 2 mins) from the separate heating belt in upstream. The whole system was maintained at their respective temperatures for 15–20 mins (growth time) and then cooled down to room temperature naturally. Argon (**Ar**) flow was maintained throughout an experiment at the rate of 300–400 sccm. The experimental conditions to prepare set of samples is shown in Table 1. Here, typically followed a process as did for MWS-1, and carried out a sulfurization for 15 mins without using WO_3_ source material and the sample is named MoS and another experiment conducted using WO_3_ alone without sulfurization, that is, after the main temperature reaching 875 °C and the system was kept for 15 mins and naturally cooled down and the sample is named MoWO.

**Table 1:** Parameters used for the preparation of MWS-1 samples are given here.

Main furnace temperature (°C)	Sulfur temperature (°C)	Growth time (mins)	Ar flow (sccm)	Sample name
875	250	15	300	MWS-1a
825	250	15	300	MWS-1b
875	250	15	400	MWS-1c
875	250	15	300	MoS^*^
875	—	15	300	MoWO^$^

*No WO_3_ is used, only sulfurization of graphene/Mo-TEM grid was carried out.

^$^No sulfur is used. Only WO_3_ was heated over which graphene/Mo-TEM grid placed face down.

### STEM characterization

Low magnification annular dark field – scanning transmission electron microscopy (ADF-STEM) images were first obtained with FEI Technai F20 to have a preliminary check over the samples and high resolution atomic-scale ADF-STEM images were captured using FEI Chemi-STEM Titan G^2^ which was equipped with a probe-side spherical aberration-corrector and operated at an acceleration voltage of 200 kV. The convergent semi-angle for illumination was set to 24 mrads with a probe current of 50–70 pA, and the collection angle was 50–100 mrads. Energy dispersive X-ray spectroscopy (EDS) was carried out on a Bruker super-X detection system.

### Data availability

The datasets generated during and/or analyzed during the current study are available from the corresponding author on reasonable request.

## Electronic supplementary material

Supplementary Information
