# Supplementary material for: Growth of ‘W’ doped molybdenum disulfide on graphene transferred molybdenum substrate
Source: Sci Rep. 2018 May 9;8:7396. doi: 10.1038/s41598-018-25796-9 (PMC5943342; doi:10.1038/s41598-018-25796-9)
Supplement: Supplementary file 1 — Supplementary Information [file 41598_2018_25796_MOESM1_ESM.doc]

**Growth of ‘W’ doped molybdenum disulfide on graphene transferred molybdenum substrate**

**Vijayshankar Asokan^1,2^, Dancheng Zhu^1^, Wei Huang^1^, Hulian Wang^1^, Wandong Gao^1^, Ze Zhang^1^, Chuanhong Jin^1, *^**

^1^State Key Laboratory of Silicon Materials, School of Materials Science and Engineering, Zhejiang University, Hangzhou, Zhejiang, 310027 China.

^2^Present address: Environmental Inorganic Chemistry, Department of Chemistry and Chemical Engineering, Chalmers University of Technology, Gothenburg, 41296, Sweden.

*Corresponding author. E-mail address: chhjin@zju.edu.cn (C. J.)

Supplementary Information


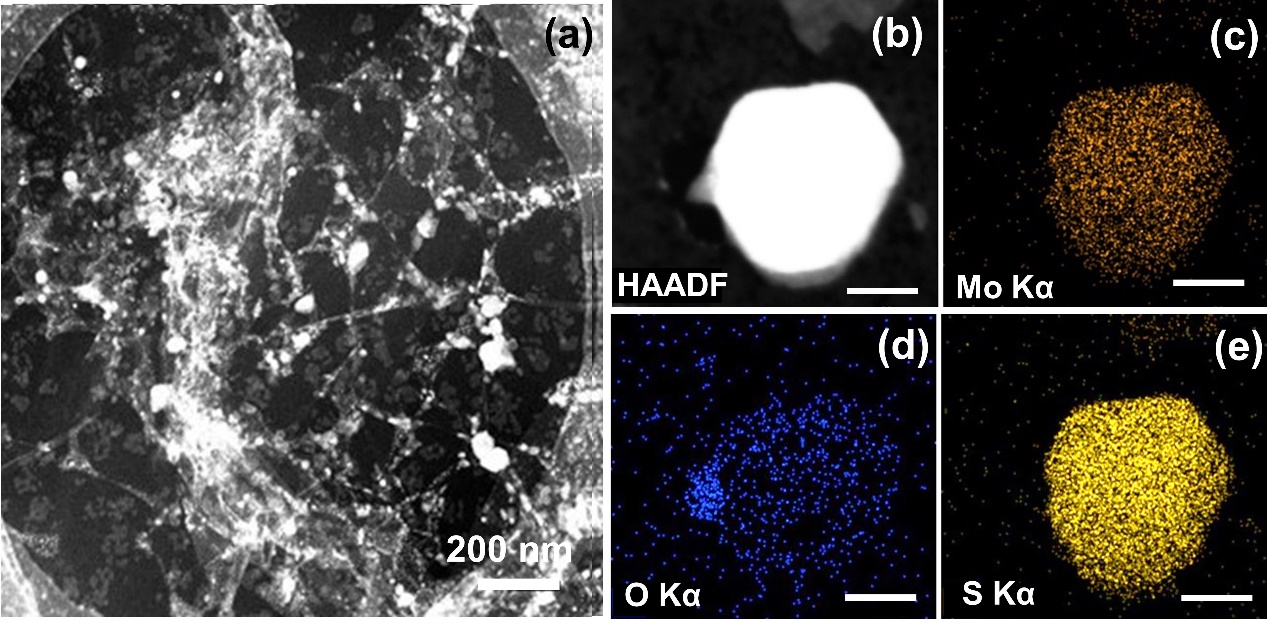


Figure S1: (a) A low magnification ADF-STEM images of MoS, and (b-e) EDS mapping, scale bar: 20 nm.


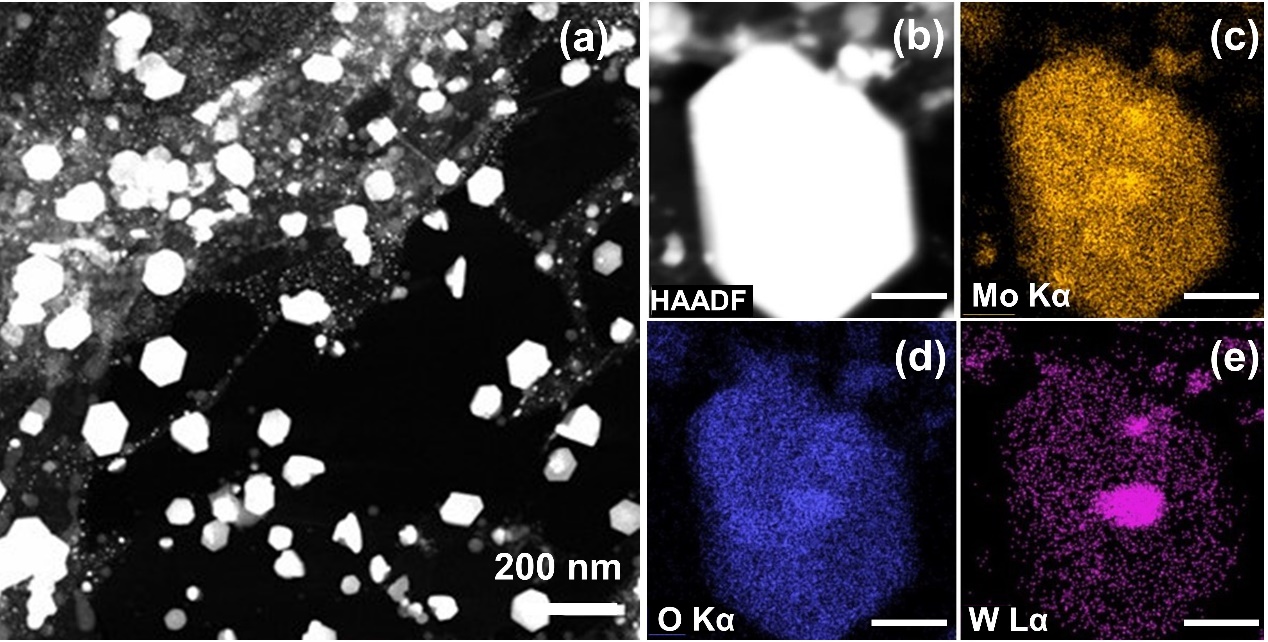


Figure S2: (a) A low magnification ADF-STEM images of MoWO, and (b-e) EDS ma ping, scale bar: 40 nm.
